# Impact of Sr Addition on Zirconia–Alumina-Supported Ni Catalyst for CO*_x_*-Free CH_4_ Production via CO_2_ Methanation

**DOI:** 10.1021/acsomega.3c08536

**Published:** 2024-02-14

**Authors:** Abdulaziz A. M. Abahussain, Ahmed S. Al-Fatesh, Yuvrajsinh B. Rajput, Ahmed I. Osman, Salwa B. Alreshaidan, Hamid Ahmed, Anis H. Fakeeha, Abdulrhman S. Al-Awadi, Radwa A. El-Salamony, Rawesh Kumar

**Affiliations:** † Chemical Engineering Department, College of Engineering, King Saud University, P.O. Box 800, Riyadh 11421, Saudi Arabia; ‡ Department of Chemistry, Indus University, Ahmedabad, Gujarat 382115, India; § School of Chemistry and Chemical Engineering, Queen’s University Belfast, Belfast, Northern Ireland BT9 5AG, U.K.; ∥ Process Development Department, Egyptian Petroleum Research Institute (EPRI), Cairo 11727, Egypt

## Abstract

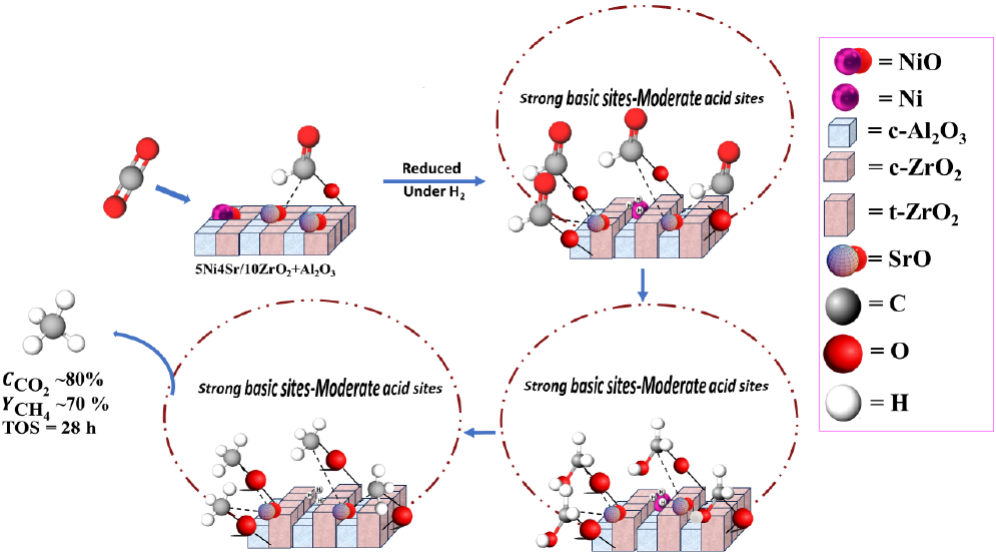

Zirconia-alumina-supported Ni (5Ni/10ZrO_2_+Al_2_O_3_) and Sr-promoted 5Ni/10ZrO_2_+Al_2_O_3_ are prepared, tested for carbon dioxide (CO_2_) methanation at 400 °C, and characterized by X-ray diffraction, X-ray photoelectron spectroscopy, surface area and porosity, infrared spectroscopy, and temperature-programmed reduction/desorption techniques. The CO_2_ methanation is found to depend on the dispersion of Nickel (Ni) sites as well as the extent of stabilization of CO_2_-interacted species. The Ni active sites are mainly derived from the reduction of ‘moderately interacted NiO species’. The dispersion of Ni over 1 wt % Sr-promoted 5Ni/10ZrO_2_+Al_2_O_3_ is 1.38 times that of the unpromoted catalyst, and it attains 72.5% CO_2_ conversion (against 65% over the unpromoted catalyst). However, increasing strontium (Sr) loading to 2 wt % does not affect the Ni dispersion much, but the concentration of strong basic sites is increased, which achieves 80.6% CO_2_ conversion. The 5Ni4Sr/10ZrO_2_+Al_2_O_3_ catalyst has the highest density of strong basic sites and the highest concentration of active sites with maximum Ni dispersion. This catalyst displays exceptional performance and achieves approximately 80% CO_2_ conversion and 70% methane (CH_4_) yield for up to 25 h on steam. The unique acidic–basic profiles composed of strong basic and moderate acid sites facilitate the sequential hydrogenation of formate species in the CO_*x*_-free CH_4_ route.

## 1 Introduction

The catalytic conversion of CO_2_ to CH_4_ represents a promising approach to reducing CO_2_ concentration in the environment and addressing the energy crisis through CH_4_ production.^1^ CH_4_ can serve as a substitute for natural gas,^2^ and it can also be used to generate electricity.^3^ CO_2_ methanation is a crucial process for future space travel as its reactants, CO_2_ and H_2_, are continuously produced from respiration and water electrolysis.^4^ The production of H_2_ from electrolysis and then the potential conversion of H_2_ and CO_2_ into methane is termed as the power-to-gas conversion process.^5^

This reaction is commonly termed as the CO_2_ methanation reaction. From a catalytic perspective, there is a preference for cost-effective Ni-based catalysts over precious metals, such as Ru, Ir, Rh, and Pd for methanation reactions.^3,6,7^ These Ni-based catalysts offer a more economical and sustainable solution for efficient CO_2_ conversion into CH_4_, making them a key focus in current research and development efforts.^8^ The interaction between CO_2_ and the catalyst site is influenced by two critical factors: surface basicity and reducibility. Surface basicity induces the CO_2_ interaction. Metallic Ni and oxygen defects are formed as a consequence of surface reduction. Oxygen defects are the sites where CO_2_ activation occurs, while metallic Ni facilitates H_2_ dissociation.^9^ The interaction between activated CO_2_ and dissociated hydrogen molecules progresses the CO_2_ hydrogenation reaction through two routes: the direct route, which does not involve CO as an intermediate, and the indirect route, which includes CO as an intermediate in the reaction mechanism. For instance, a zirconia-supported Ni catalyst prepared by the plasma decomposition method exhibits more exposed Ni (111) sites (for H_2_ dissociation) with fewer defects.^10^ Indeed, the presence of fewer defects might not be sufficient to adequately stabilize the formate species, leading to its further decomposition into CO, which is subsequently hydrogenated into CH_4_ through an indirect route. Zirconia-supported Ni catalysts prepared through the thermal decomposition method introduce a suitable number of defects, thereby ensuring the effective stabilization of formate species. Consequently, this facilitates the sequential hydrogenation of formate into CH_4_ (without the formation of CO) through a direct route. This emphasizes the influence of catalyst preparation methods on the reaction mechanism in the CO_2_ methanation reaction.

Zirconia has oxygen-endowing capacity and yttria-zirconia has thermal stability as well.^10^ Notably, zirconia-supported Ni catalysts, Sr-modified Ni-ZrO_2_, and lanthana–zirconia-supported Ni catalysts have been recognized for achieving 100% CH_4_ selectivity through the direct methanation route.^11−13^ In alumina–zirconia-supported Ni catalysts (Al/Zr = 1/10 molar ratio), the H_2_ dissociation capacity over Ni was enhanced.^14^ In the same way, enhanced basicity was observed over Sr-doped Ni-based catalysts supported over the WO_3_+ZrO_2_ catalyst, which showed 90% CO_2_ conversion and 84% CH_4_ yield up to 300 min at 350 °C.^14,15^ Considering the catalyst cost, the cheap alumina and silica supports have consistently garnered significant interest compared to other supports, such as yttria, zirconia, lanthana, and ceria. Among the alumina and silica, silica has a lower Ni dispersion ability, which caused inferior catalytic activity toward the CO_2_ methanation reaction.^16^ The utilization of 20 wt % Ni supported over Al_2_O_3_ resulted in 75% CO_2_ conversion and 96% CH_4_ selectivity.^17,18^ However, in the case of an alumina-supported Ni catalyst, Ni tends to migrate into the alumina lattice, thereby affecting both hydrogen dissociation and hydrogenation of CO_2_. To mitigate this issue, the incorporation of ZrO_2_ alongside alumina prevents the diffusion of Ni into the alumina lattice, resulting in improved stability in the CO_2_ methanation reaction.^19^ Moreover, the addition of ZrO_2_ into the Ni–Al binary hydrotalcite was found to increase the number of basic sites as well as surface oxygen vacancy.^20^ Ni-based nanocatalysts with 15 wt % Zr contents prepared from NiZrAl-layered double hydroxide precursors were observed to enrich the catalyst surface with ‘Zr^3+^–oxygen vacancy’ species, which are crucial for CO_2_ activation.^21^ The addition of silica to alumina in Ni-based catalysts shows inferior catalytic performance in the CO_2_ methanation reaction.^22^ However, when Si/Al = 0.5, the Ni/Al_2_O_3_–SiO_2_ catalyst exhibited improved reducibility and high dispersion of metallic Ni. This favorable configuration resulted in notable performance, achieving an 82.38% CO_2_ conversion and 98.19% CH_4_ selectivity at 350 °C.^23^ The presence of 15 wt % MnO_2_ in 10 wt % NiO-Al_2_O_3_ catalyst caused enhanced reducibility, higher CO_2_ adsorption, enhanced dispersion of active sites, and stable catalytic activity (84% CO_2_ conversion and 98% CH_4_ selectivity) up to 800 min.^24^ The incorporation of 10 wt % Co into Al_2_O_3_-supported 10 wt % Ni catalyst had a positive impact, enhancing both Ni dispersion and reducibility, which turned into an improved performance of >65% CO_2_ conversion (as compared to the 60% CO_2_ conversion over 10Ni/Al_2_O_3_) and 95% CH_4_ selectivity at 350 °C.^17^ Using CaO along with Al_2_O_3_ (Ca/Al = 1/2 mol ratio) as support for Ni-based catalysts was found to enhance the reducibility and activity (∼80% CO_2_ conversion and 99% CH_4_ selectivity) for up to 12 h.^25^ 3 wt % Co-promotional addition over 10 wt % Ni supported on CaO-Al_2_O_3_ (Ca/Al = 1/2) further induced a higher degree of reducibility and formation of more active sites, which resulted in 83% CO_2_ conversion and 99% CH_4_ selectivity constantly up to 900 min.^26^ Introducing 5 wt % Fe into 30 wt % Ni/Al_2_O_3_ resulted in improved reducibility, significantly enhancing CO_2_ conversion, reaching approximately 70% CO_2_ conversion (∼99% CH_4_ selectivity) at 35 °C.^27^ The addition of lanthana to the Ni/Al_2_O_3_ catalyst introduced moderate basic sites and significantly improved both Ni dispersion and reducibility.^28^ The interface between LaO_*x*_ and Ni crystallite may be accountable for catalytic activity toward CO_2_ methanation.^29^ The incorporation of lanthana into silica-modified alumina enhances the catalyst’s basicity, leading to a significant improvement in CH_4_ selectivity, with almost 100% CH_4_ selectivity (84% CH_4_ yield) achieved at a reaction temperature of 300 °C.^19^ The stoichiometric ratio of MgO, NiO, and Al_2_O_3_ in hydrotalcite-like structures led to a strong interaction of NiO with the hydrotalcite matrix, resulting in catalytic inferiority. However, with an increase in Ni loading to 42.5 wt % in hydrotalcite, the interaction of NiO with the matrix weakened, leading to higher catalytic activity with 82% CO_2_ conversion and 99% CH_4_ selectivity achieved at 300 °C. Furthermore, the addition of yttria to the alumina-supported Ni catalyst was found to enhance reducibility, increase CO_2_ uptake, and improve the interaction between Ni and the Al_2_O_3_ support.^30^ Sr incorporation in Ni/Al_2_O_3_ optimizes the crystalline size.^31^ It was observed that alumina-supported SrO exhibits higher CO_2_-carrying capacity compared to unsupported SrO, which makes promising material for CO_2_ capture and catalysis.^15,31−33^ The incorporation of Sr into alumina-supported Rh–Mn catalyst has been observed to impact the degree of reduction and CO chemosorption, leading to enhanced CO_2_ conversion (73%) and 40% CH_4_ yield at 210 °C.^34^ Moreover, the use of zirconia-based support has been found to provide resistance to the sintering of SrO during CO_2_ sorption at high temperatures.^35^

After an extensive literature survey, we speculate that the strontium-promoted alumina-supported Ni catalyst may be excellent for the CO_2_ methanation reaction. But it may encounter sintering of SrO at high temperatures and diffusion of Ni into the alumina lattice.^19,32,35^ Using zirconia proportion along with alumina can provide resistance to SrO sintering, and it can prevent the diffusion of Ni into alumina lattice. In this study, we have developed a novel strontium-promoted zirconia-alumina-supported Ni catalyst for CO_2_ methanation. Prior to the reaction, the catalyst underwent reduction, and various characterizations, including X-ray diffraction, surface area and porosity analysis, CO_2_-temperature-programmed reduction, infrared spectroscopy, and transmission electron microscopy, were conducted to assess its properties. The fresh catalysts were also characterized and discussed for comparison purposes. XPS analysis provided valuable insights into the oxidation states of the elements within the catalyst. By closely correlating the characterization results with catalytic activity, we aim to establish a well-suited catalyst for industrial CO_2_ methanation in the near future. The findings of this study pave the way for the development of efficient and sustainable catalysts to address the challenges of CO_2_ methanation.

## 2 Experimental Section

### 2.1 Material

Ni (NO_3_)_2_.6H_2_O (Fisher, Germany), Sr (NO_3_)_3_.6H_2_O (Fisher, Germany), and 10ZrO_2_+γ-Al_2_O_3_ microspheres (dp = 400–500 μm; Daiichi Kigenso Kagaku Kogyo Co., Ltd.) were used to prepare the catalysts. As per the specification of 10ZrO_2_+Al_2_O_3_ (from Daiichi Kigenso Kagaku Kogyo Co., Ltd.), the 10ZrO_2_+Al_2_O_3_ support has 126 m^2^/g surface area and 50% of particles in the catalyst are smaller than 60.3 μm (*D*_50_ = 60.3 μm). It has tetragonal ZrO_2_ and cubic γ-Al_2_O_3_ phases. The concentration of basic sites and acidic sites over the 10ZrO_2_+Al_2_O_3_ support is lower than ZrO_2_.

### 2.2 Catalyst Preparation

5 wt % nickel and 1–4 wt % strontium are incorporated into 10ZrO_2_+γ-Al_2_O_3_ by an impregnation method. Nickel nitrate (equivalent to 5 wt % Ni) and strontium nitrate solutions (equivalent to 1–4 wt %) are added to the 10ZrO_2_+γ-Al_2_O_3_ support while stirring and heating. The mixture is held in an 80 °C bath with moderate stirring until it evaporates. It is then dried overnight at 110 °C before being calcined for 30 min at 450 °C (heating ramp 1 °C/min). The prepared catalysts are named as 5Ni/10ZrO_2_+Al_2_O_3_, 5Ni1Sr/10ZrO_2_+Al_2_O_3_, 5Ni2Sr/10ZrO_2_+Al_2_O_3_, 5Ni3Sr/10ZrO_2_+Al_2_O_3_, and 5Ni4Sr/10ZrO_2_+Al_2_O_3_.

### 2.3 Catalysts Characterization

The X-ray diffraction study of the catalyst sample is carried out with a Miniflex Rigaku diffractometer (Rigaku, Saudi Arabia) using a Cu_Kα_ source (λ= 1.54056) operated at 40 kV and 40 mA. The step size and scanning range of 2θ for analysis were set to 0.01 and 5–100, respectively. The peak search profile is adjusted at a minimum significance of 2, minimum tip width of 2θ = 0.01°, maximum tip width 2θ = 1°, and peak base width of 2° under the minimum second derivative method. Peak search and matching are carried out at a search depth of 10 and a minimum scale factor of 0.1. The diffraction patterns of the sample are matched with the JCPDS database for phase analysis. The oxidation state of elements is determined by X-ray photoelectron spectroscopy (XPS) using a Thermo-fisher Scientific instrument (USA) through an Al_Kα_ excitation source and 20 eV pass energy. The N_2_ adsorption–desorption profile against relative pressure (P/P_o_), surface area, pore volume, and pore diameter of the catalyst sample was obtained from Micromeritics Tristar II 3020 instrument (Micromatics, USA). The surface area is estimated by the Brunauer–Emmet–Teller equation, whereas pore size distribution is estimated by the nonlocal density function model. The reducibility, basicity, and acidity profiles are studied by H_2_-temperature-programmed reduction (H_2_-TPR), CO_2_-temperature-programmed desorption and NH_3_-temperature-programmed desorption by using Micromeritics Autochem II 2920 (Micromatics, USA) and thermal conductivity detector (TCD). For H_2_-TPR, 70 mg of the sample was heated to 900 °C (at a heating ramp of 10 °C/min) under 10% H_2_/Ar gas feed (flow rate 40 mL/min). After the interaction of the gas feed with a surface, H_2_O is formed, which is removed using a cold trap. The Ni dispersion was evaluated by a H_2_ chemisorption study using a BELCAT II Catalyst Analyzer. After in situ reduction of the sample at 700 °C for 1 h in 5% H_2_–N_2_ mixture (30 mL/min), it was cooled down in the inert atmosphere to 100 °C, and pulses of 5% H_2_–N_2_ mixture of known volume were injected until the metal surface was saturated and no further H_2_ uptake was observed. For TPD, 70 mg of the sample was cleaned using helium flow at 200 °C for 1 h. Then, it was fed with a mixture of 10% CO_2_ (or 10% NH_3_)/He gas feed (flow rate 30 mL/min) at 50 °C for 30 min. CO_2_ or NH_3_ was desorbed with an increasing temperature of up to 900 °C. The change in conductivity due to the consumption of H_2_ in TPR or desorption of gases in TPD over the catalyst surface was recorded by a temperature conductivity detector (TCD). Fourier transform infrared (FTIR) spectra of catalyst samples were taken by Prestige-21 SHIMADZU. The catalyst morphology was observed by 120 kV JEOL JEM-2100F (Akishima, Japan) transmission electron microscope (TEM).

### 2.4 Catalytic Activity Test

**Figure 1:**
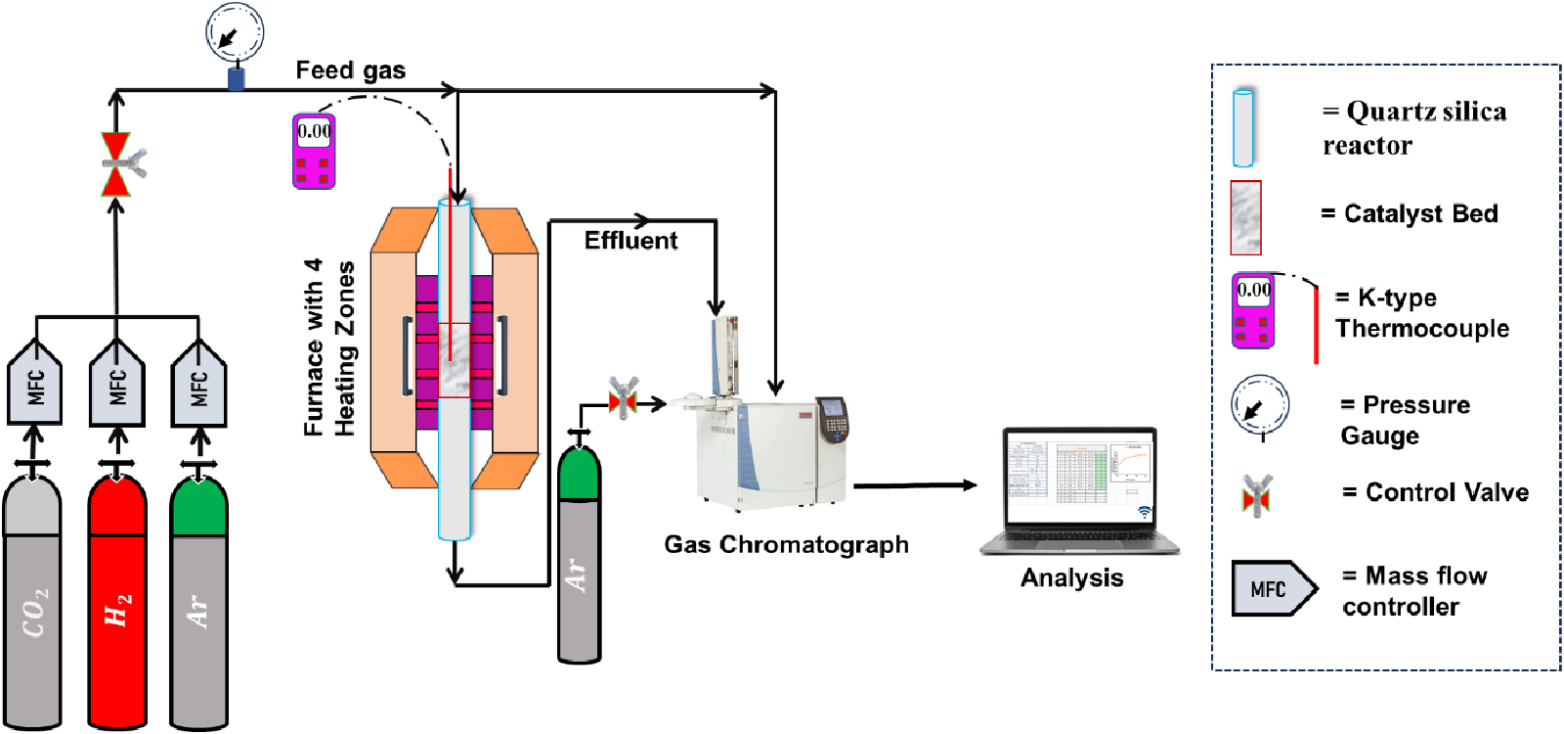
Systematic diagram for experimental setup for the CO_2_ methanation reaction.

The reactor set for the CO_2_ methanation reaction is shown in Figure 1. The CO_2_ methanation reaction is carried out over a packed catalyst (2 g of catalyst diluted with silicon carbide up to 5 cm bed height) in a tubular fixed bed quartz silica reactor (length = 50 cm, inner diameter = 13 mm, catalyst bed volume 5 cm^3^). The temperature for the reaction is given by a peripheral programmable electric furnace, and the temperature of the catalyst bed is monitored by a K-type thermocouple, which is placed axially in the middle of the catalyst bed (as shown in Figure 1). First, the catalyst is reduced under H_2_ (flow rate 30 mL/min) for 2 h at 700 °C, and then the reactor is cooled down. Furthermore, for the methanation reaction, CO_2_: H_2_: Ar (1:4:5 volume ratio) gas feed is passed over the reduced catalyst at 6000 ccg^–1^h^–1^ GHSV and a 400 °C reaction temperature. The reaction products and unconverted feed gases from the reactor were evaluated quantitatively by using an online GC (GC-Shimadzu 2014) equipped with molecular sieve 5A and Porapak Q column and thermal conductivity detector (TCD). The carbon dioxide conversion () and methane yield () are calculated as shown below:^19^
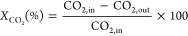
1
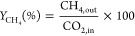
2

## 3 Results and Discussion

### 3.1 Characterization Results

**Figure 2:**
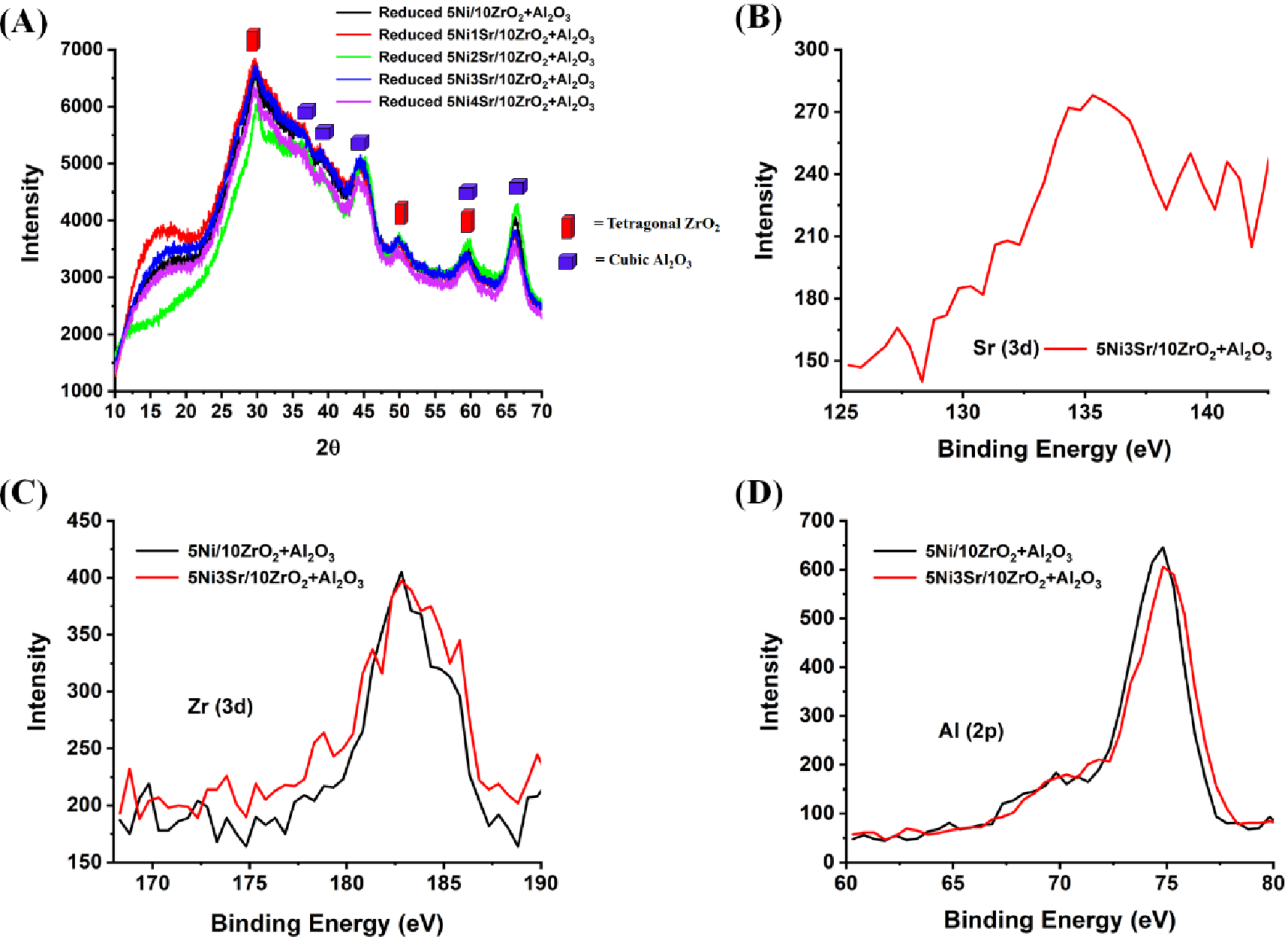
(A) X-ray diffraction patterns of the reduced Ni/10ZrO_2_+Al_2_O_3_ and reduced NixSr/10ZrO_2_+Al_2_O_3_ (*x* = 1–4) catalyst system. (B) Sr (3d) XPS spectra, (C) Zr (3d) XPS spectra, and (D) Al (2p) XPS spectra.

**Figure 3:**
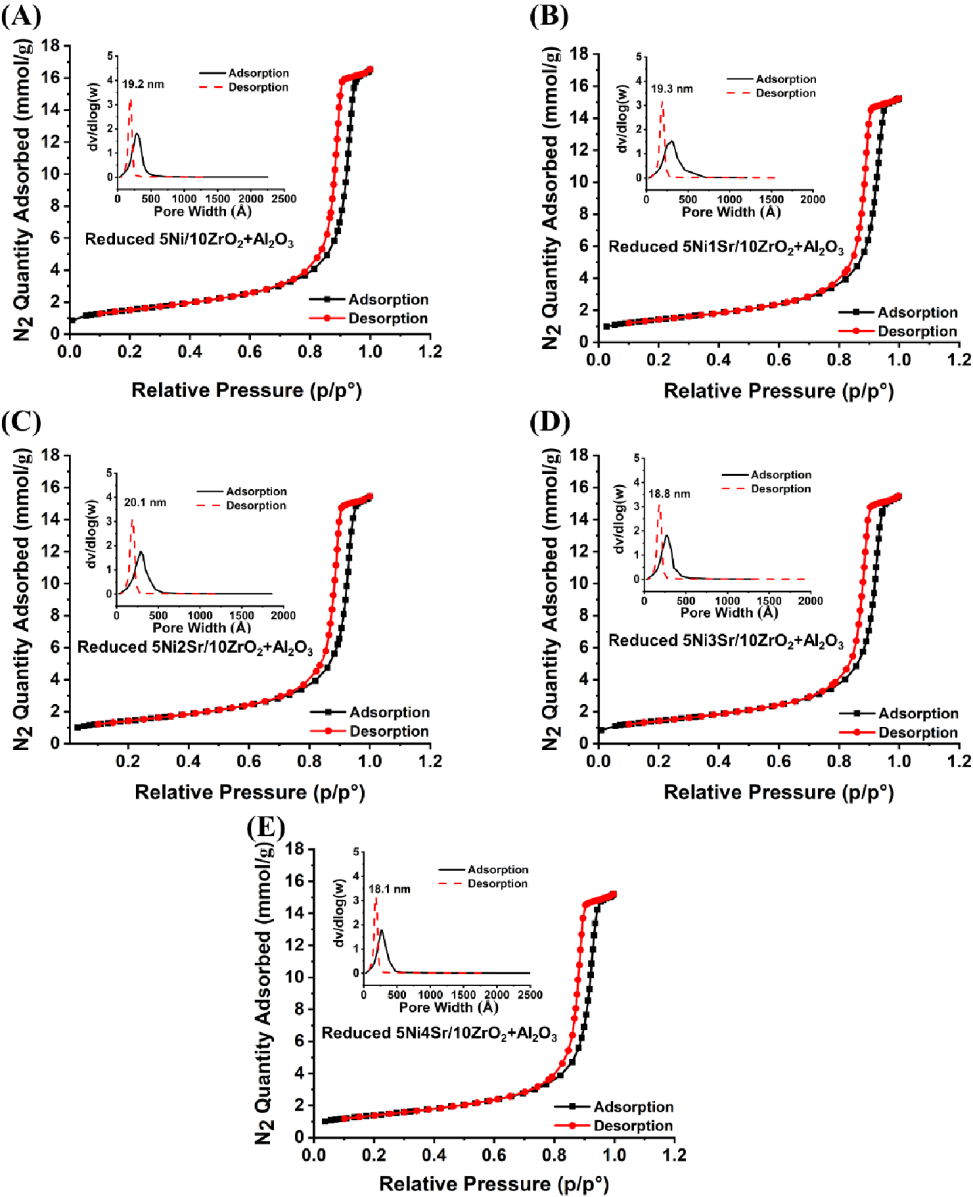
(A–E) Nitrogen sorption isotherm of reduced 5NixSr/10ZrO_2_+Al_2_O_3_ (*x* = 0–4 wt %) catalyst system. The inset figure shows the porosity distribution of the reduced 5NixSr/10ZrO_2_+Al_2_O_3_ (*x* = 0–4 wt %) catalyst system.

Before the onset of the CO_2_ methanation reaction, catalyst reduction was performed. Hence, identifying phase distribution, surface area, basicity, and acidity profiles over the reduced catalyst system is essential herein. Figure 2A displays the X-ray diffraction results for the reduced Ni/10ZrO_2_+Al_2_O_3_ and reduced NixSr/10ZrO_2_+Al_2_O_3_ (*x* = 1–4) catalytic systems. All the reduced catalysts exhibited similar diffraction patterns, indicating the presence of tetragonal ZrO_2_ phase (at 2θ = 29.82, 49.76, 59.72; JCPDS reference number 00-024-1164) and cubic Al_2_O_3_ phase (at 2θ = 36.61, 39.07, 45.10, 59.72, 66.35; JCPDS reference number 00-004-0858). Notably, the diffraction pattern of the reduced catalyst system appears to be much more intense compared with the fresh catalyst system (Figure S1). The fresh catalyst system has the cubic ZrO_2_ phase (at 2θ = 30.29°, 50.57°, 60.48°; JCPDS reference number: 00-027-0997), whereas the reduced catalyst has the tetragonal ZrO_2_ phase (Figure S1). In our study, the sample undergoes reduction at 600 °C. It has been reported that the energy difference between tetragonal ZrO_2_ and cubic ZrO_2_ increases with increasing temperature, and at high temperatures, the tetragonal phase becomes more stable than the cubic phase. Interestingly, in the fresh Sr-doped catalyst, the presence of the orthorhombic SrCO_3_ phase (at 2θ = 25.1°, 36.4°, 45.5°, 49.9°; JCPDS reference number: 00-001-0556) is observed.^36,37^ However, in the reduced catalyst system, this phase is not detected, indicating that the reduction process leads to the disappearance or transformation of the SrCO_3_ phase. In both the fresh and reduced catalyst systems, Ni-related phases are not detected by XRD, indicating that Ni is finely dispersed beyond the detection limit of this technique. Sr (3d) XPS spectra at 134.2–135.9 eV binding energy, Al (2p) XPS spectra at 74.81 eV binding energy, and Zr (3d) XPS spectra at 182.9 eV binding energy confirms the +2 oxidation state of Sr, + 3 oxidation state of Al, and +4 oxidation state of Zr, respectively (Figure 2B–D).^38−40^ This information is valuable in understanding the chemical composition and surface properties of the catalyst, which play a crucial role in its catalytic activity during CO_2_ methanation. All fresh and reduced catalysts show type IV adsorption–desorption isotherms with an H1 hysteresis loop, which confirms the presence of the model of mesopores (Figures 3 and S2).

The surface area, pore volume, and pore diameters of both Sr-promoted and unpromoted catalyst systems show similar values (Table 1). Before the methanation reaction, the catalyst is reduced, and thus, the surface parameters of the reduced catalyst system accurately represent the surface area and porosity of the catalyst before the reaction. Interestingly, the surface area and the pore volume of the reduced 5Ni/10ZrO_2_+Al_2_O_3_ catalyst remain unchanged compared to the fresh catalyst, whereas in the Sr-promoted catalyst system (5NixSr/10ZrO_2_+Al_2_O_3_; *x* = 1–4 wt %), these parameters are decreased upon reduction of the catalyst (Figure S2). This observation suggests that Sr promotion may influence the surface properties of the catalyst upon reduction. The XRD analysis revealed that the diffraction pattern of the orthorhombic SrCO_3_ phase disappears upon reduction. This indicates that the orthorhombic SrCO_3_ phase undergoes restructuring and reduction during the reduction process. The d(*V*)/d(log*W*) vs *W* plot shows a unimodal pore size distribution in all reduced catalyst systems, with the major pore volume being occupied by pores of 18–20 nm in all spent catalysts. This indicates that the reduction process affects the pore structure of the catalyst, leading to a similar unimodal pore size distribution dominated by pores of a specific size range in all the spent catalysts. These findings provide valuable insights into the structural changes occurring in the catalysts during reduction and their implications for catalytic activity in the CO_2_ methanation reaction.

**Table 1:** Surface Area, Pore Volume, Pore Diameter, Ni Dispersion, CO_2_ Conversion, and CH_4_ Yield of 5Ni/10ZrO_2_+Al_2_O_3_ and 5NixSr/10ZrO_2_+Al_2_O_3_ (*x* = 0–4 wt %) Catalysts^a^

	surface area (m^2^/g)		pore volume (cm^3^/g)		pore size (nm)				
catalyst sample	Fr.	Red.	Fr.	Red.	Fr.	Red.	Ni dispersion (mmol/g)		
5Ni/10ZrO_2_+Al_2_O_3_	125	121.5	0.61	0.60	15.8	16.1	0.059	65	61
5Ni1Sr/10ZrO_2_+Al_2_O_3_	124.5	113.5	0.60	0.56	15.6	15.9	0.082	72.5	63.6
5Ni2Sr/10ZrO_2_+Al_2_O_3_	123.9	114.2	0.61	0.56	15.6	15.7	0.081	80.6	71.5
5Ni3Sr/10ZrO_2_+Al_2_O_3_	122.8	114.3	0.61	0.57	15.5	15.5	0.096	82.5	73.4
5Ni4Sr/10ZrO_2_+Al_2_O_3_	122.0	111.0	0.61	0.56	15.5	15.6	0.1	84.3	75.9

a Fr. = Fresh catalyst, Red. = Reduced catalyst,  = CO_2_ conversion,  = CH_4_ yield.

The infrared spectra of the reduced catalyst systems showed relatively intense absorption bands corresponding to physically adsorbed CO_2_ species at 2349 cm^–1^,^41^ as well as format species at 2850 and 2925 cm^–1^,^41^ which are more prominent compared to the fresh catalyst (Figure S3).

**Figure 4:**
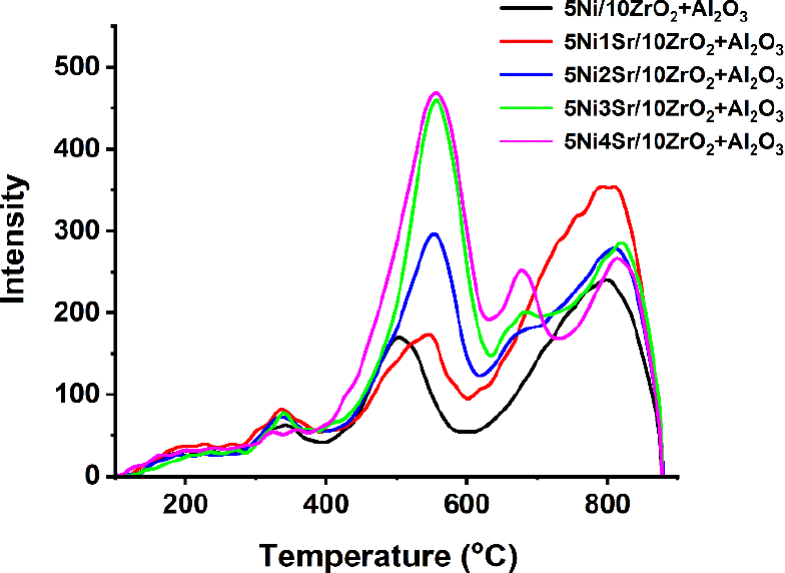
H_2_-temperature-programmed reduction study of 5Ni/10ZrO_2_+Al_2_O_3_ and 5NixSr/10ZrO_2_+Al_2_O_3_ (x = 1–4) catalysts.

The temperature-programmed studies are carried out to understand the reducibility profile of fresh catalyst, the basicity profile of spent catalyst, and the acidity profile of the spent catalyst. The H_2_-TPR study confirms consumption of hydrogen in 400–630 °C and 630–1000 °C temperature range. The former signifies the amount of ‘NiO-species which interacted with support with moderate interaction’, while the latter is attributed to the amount of ‘NiO-species which interacted strongly with the support’ (Figure 4). Here, prior to the CO_2_ methanation reaction, catalyst reduction was carried out at 700 °C under hydrogen. So up to 700 °C, mostly ‘moderately interacting NiO-species’ are reduced to metallic Ni. Furthermore, metallic Ni becomes the center for hydrogen dissociation during the CO_2_ methanation reaction. Upon 1 wt % addition of Sr over 5Ni/10ZrO_2_+Al_2_O_3_, the amount of ‘moderately interacted NiO-species’ is not affected. It is noticeable that upon increased loading of 1–4 wt % Sr over 5Ni/10ZrO_2_+Al_2_O_3_, the amount of ‘moderately interacted NiO-species’ is grown, and these NiO-species surges ‘Ni’ active sites upon reduction. The result of H_2_-chemosorption is in line with the H_2_-TPR results. The dispersion of metallic Ni over different catalysts is observed in the following order: 5Ni4Sr/10ZrO_2_+Al_2_O_3_ (0.1 mmol/g) > 5Ni3Sr/10ZrO_2_+Al_2_O_3_ (0.096 mmol/g) > 5Ni2Sr/10ZrO_2_+Al_2_O_3_ (0.081 mmol/g) ∼ 5Ni1Sr/10ZrO_2_+Al_2_O_3_ (0.082 mmol/g) > 5Ni/10ZrO_2_+Al_2_O_3_ (0.059 mmol/g).

Incorporating zirconia into alumina has been reported to increase the total acidity of the resulting zirconia–alumina composite compared to that of pure alumina. This increased acidity is valuable in catalysis, as it can facilitate various acid-catalyzed reactions and improve the overall catalytic performance of the material.^42^ Cai et al. also reported the presence of weak basic sites over alumina–zirconia-supported Ni catalysts.^19^ Over the current 10ZrO_2_+Al_2_O_3_ support, the concentration of acid sites and basic sites is reported to be lower than that of ZrO_2_ alone. Adding basic SrO or Sr (OH)_2_ to an acidic zirconia–alumina-supported Ni catalyst can potentially weaken the acid profile on the catalyst surface. Previous studies have shown that SrCO_3_ can enhance surface acidity over SrTiO_3_.^43^ The formation of SrO enhances the basicity of the catalyst, while SrCO_3_ may contribute to the acidity of the system. Overall, the acid–base profile of the catalyst plays a crucial role in directing the CO_2_ methanation reaction. The balance between acidic and basic sites on the catalyst surface will determine its catalytic activity and selectivity for CO_2_ methanation.

**Figure 5:**
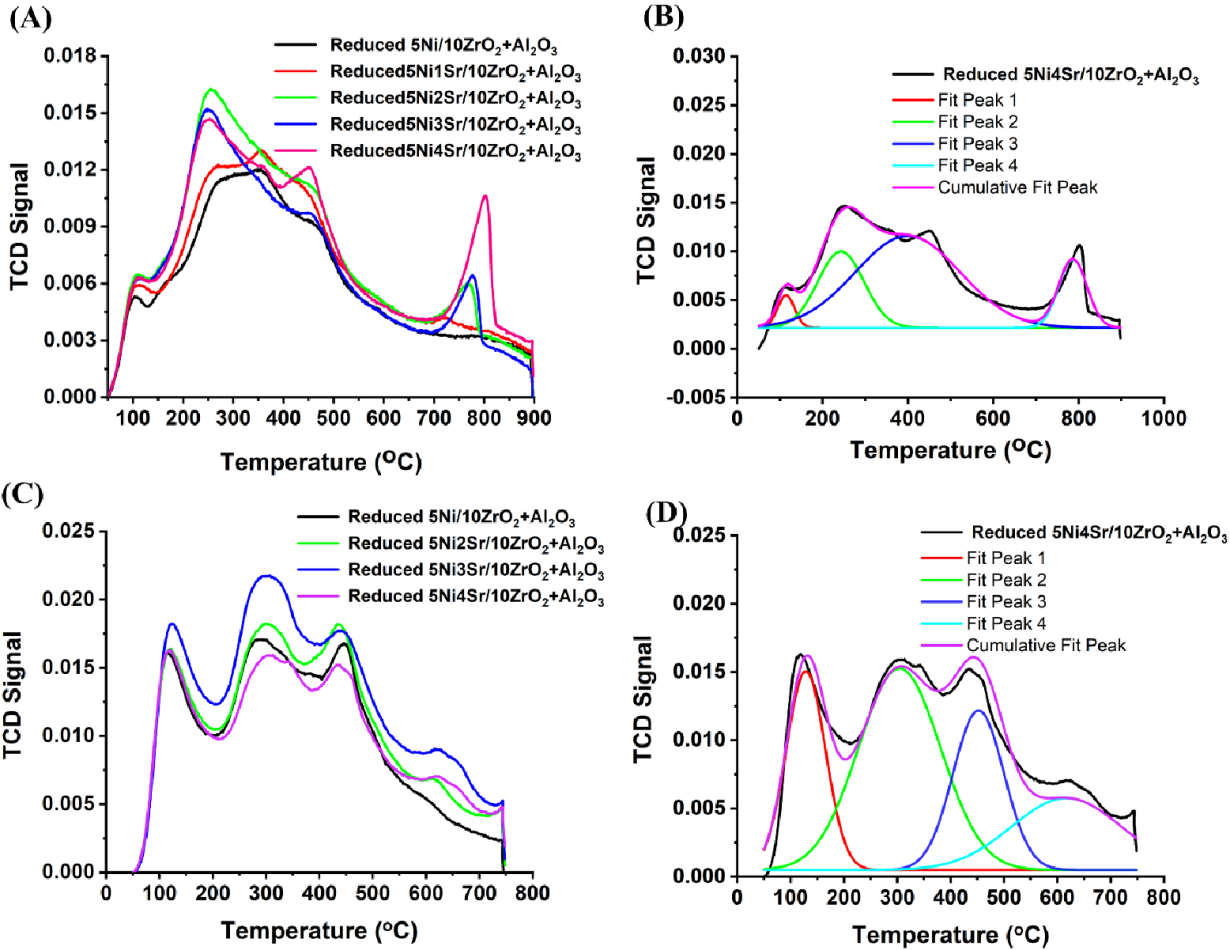
(A) CO_2_-temperature-programmed desorption profile of reduced 5NixSr/10ZrO_2_+Al_2_O_3_ (*x* = 0–4 wt %) catalyst system, (B) peak fitting of CO_2_-TPD of the 5Ni4Sr/10ZrO_2_+Al_2_O_3_ catalyst, (C) NH_3_-temperature-programmed desorption profile of reduced 5NixSr/10ZrO_2_+Al_2_O_3_ (*x* = 0–4 wt %) catalyst system, (D) peak fitting of NH_3_-TPD of the 5Ni4Sr/10ZrO_2_+Al_2_O_3_ catalyst.

The basic profile of both fresh and reduced 5NixSr/10ZrO_2_+Al_2_O_3_ catalysts was investigated by CO_2_ desorption (Figures 5A,B and S4). The fresh 5Ni/10ZrO_2_+Al_2_O_3_ catalyst exhibited a diffused desorption peak around 100 °C, corresponding to a weak basic site (CO_2_ adsorbed over surface hydroxyl-generating HCO_3_^–^ species) and a broad peak in the region of 250–450 °C, attributed to moderate strength basics sites (CO_2_ adsorbed over surface oxide ion).^44−46^ After reduction, the CO_2_-TPD profile of the reduced catalyst represents the actual basic sites present on the catalyst surface. The reducible surface hydroxyl (constituting weak basic sites) and reducible surface oxide ion (constituting moderate basic sites) are reduced during this process, leading to a decrease in the intensity of basic sites on the reduced catalyst compared to the fresh catalyst.

In the fresh Sr-doped 5Ni/10ZrO_2_+Al_2_O_3_ catalyst, CO_2_ desorption peaks are observed at 600 and 800 °C, indicating the presence of strontium-related/induced structures (Figure S4). The interaction between basic surface oxygen (including Sr–O) and CO_2_ was found to generate ‘bonded carbonate species,’ which is decomposed at about 600 °C.^46^ Zhao et al. also claimed that the CO_2_ desorption peak, around 600 °C, is for decomposing highly dispersed SrCO_3_ species.^47^ Interestingly, this peak was not observed in the reduced catalyst, indicating that it decomposes under H_2_ steam during reduction at 700 °C. As a result, this basic site does not exist after the reductive treatment of the catalyst. The disappearance of the orthorhombic SrCO_3_ phase in XRD upon reduction supports this finding.

Another CO_2_ desorption peak at around 800 °C is observed in both fresh and reduced catalysts, and its intensity increases with increasing Sr loading. Ghorbaei et al. reported effective CO_2_ diffusivity in the SrCO_3_ layer at >800 °C.^47^ Although no other crystalline peaks of Sr-related compounds were found in the XRD of the reduced 5NixSr/10ZrO_2_+Al_2_O_3_ (*x* = 1–4) catalyst, an organization of amorphous strong basic sites may be present over the Sr-promoted catalyst, constituted by −Sr–O– and −C–O– like species. This suggests that the addition of Sr promotes the formation of strong basic sites on the catalyst surface, which may play a crucial role in guiding the CO_2_ methanation reaction.

To investigate the surface acidity profile, NH_3_-TPD of both fresh and reduced 5NixSr/10ZrO_2_+Al_2_O_3_ catalysts was conducted (Figures 5C,D and S5). The unpromoted catalyst exhibited a desorption peak of ammonia in a low-temperature region (∼100 °C) for physisorbed NH_3_ and a peak at ∼300 °C for chemosorbed NH_3_, indicating the presence of weak acid sites (Figure S5A).^48^ In contrast, the Sr-promoted 5Ni/10ZrO_2_+Al_2_O_3_ catalyst showed an additional peak in the high-temperature region (600–700 °C) for strong acid sites.^49^ It indicates that the reduction peak of about 600 °C is related to acidity borne by Sr species. Since the CO_2_ methanation reaction was performed over a reduced catalyst, the acid profile over the reduced catalyst reflects the actual acid sites during the reaction. The weak acid sites are associated with the acidity provided by the surface hydroxyl groups. After the catalyst reduction, the reducible surface hydroxyl groups are converted to water, leading to a decrease in the total surface hydroxyl concentration on the catalyst surface. As a result, when NH_3_-TPD is conducted with the reduced catalyst system, the peak intensity at around ∼300 °C (for weak acidity) is decreased compared to the fresh catalyst system (Figures 5B and S5B–G). Additionally, over the reduced Sr-promoted 5Ni/10ZrO_2_+Al_2_O_3_ catalyst, the intensity of the strong acid site at around 650 °C is reduced, while a new desorption peak at about 450 °C for moderate-strength acid sites is observed (compared with the fresh catalyst). The new NH_3_ desorption peak about 450 °C is also observed over reduced nonpromoted catalyst. It means that the acid sites reflecting around 450 °C in the NH_3_-TPD profile are not due to Sr species. It can be correlated to moderate strength acid sites, which are created after reduction of the catalyst.

**Figure 6:**
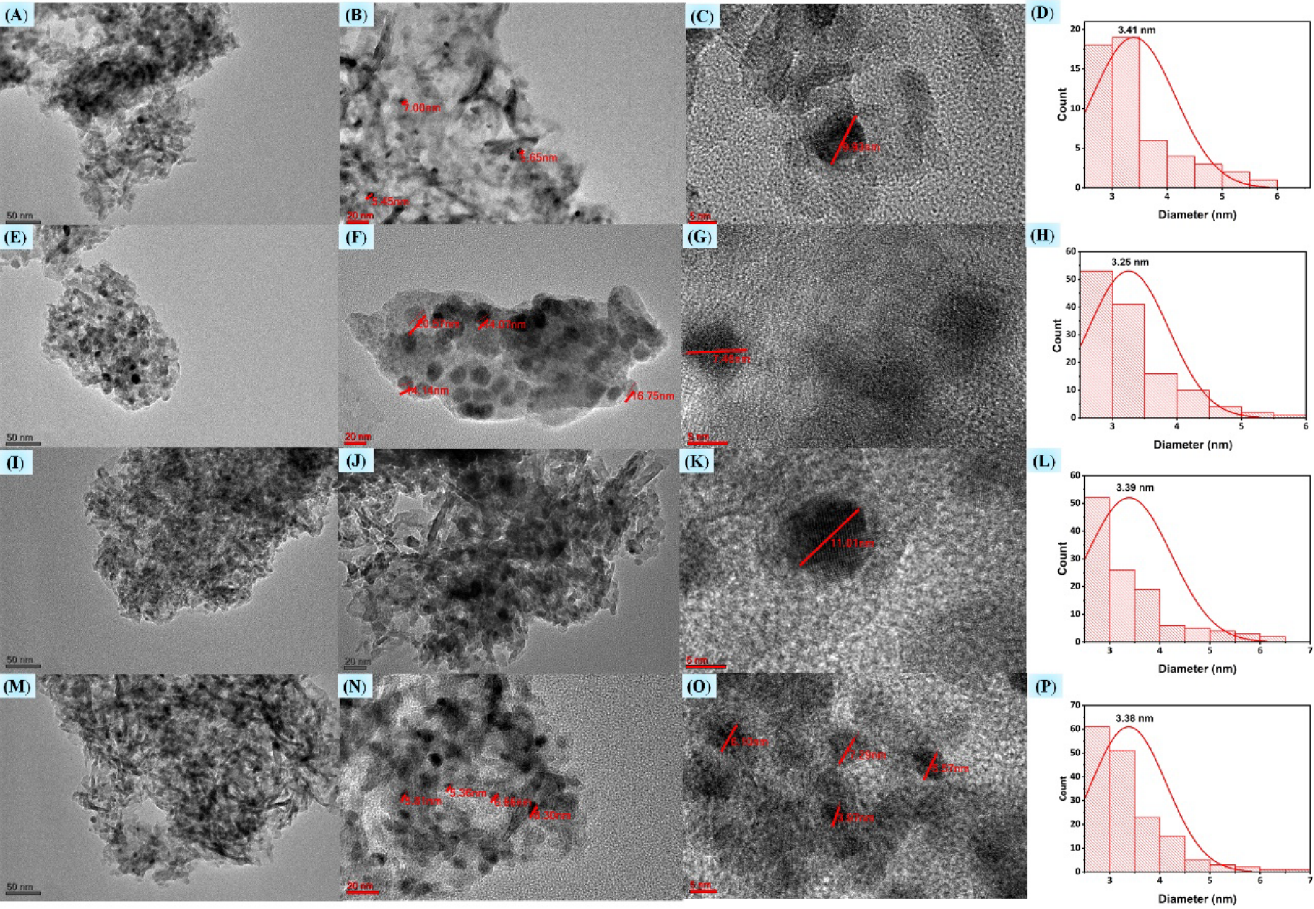
TEM images of (A–C) reduced 5Ni/10ZrO_2_+Al_2_O_3_ at 50, 20, and 5 nm scale, (D) particle size distribution of reduced 5Ni/10ZrO_2_+Al_2_O_3_, (E–G) spent 5Ni/10ZrO_2_+Al_2_O_3_ at 50, 20, and 5 nm scale, (H) particle size distribution of spent 5Ni/10ZrO_2_+Al_2_O_3_, (I–L) reduced 5Ni4Sr/10ZrO_2_+Al_2_O_3_ at 50, 20, and 5 nm scale, (L) particle size distribution of reduced 5Ni4Sr/10ZrO_2_+Al_2_O_3_, (M–P) spent-5Ni4Sr/10ZrO_2_+Al_2_O_3_ at 50, 20, and 5 nm scale, (P) particle size distribution of spent 5Ni4Sr/10ZrO_2_+Al_2_O_3_.

TEM images of reduced and spent 5Ni/10ZrO_2_+Al_2_O_3_ and 5Ni4Sr/10ZrO_2_+Al_2_O_3_ catalysts are shown in Figure 6. The particle sizes of both catalytic systems remain consistent, ranging from 3.28 to 3.41 nm over reduced and spent catalysts.

### 3.2 Catalytic Activity Results and Discussion

**Figure 7:**
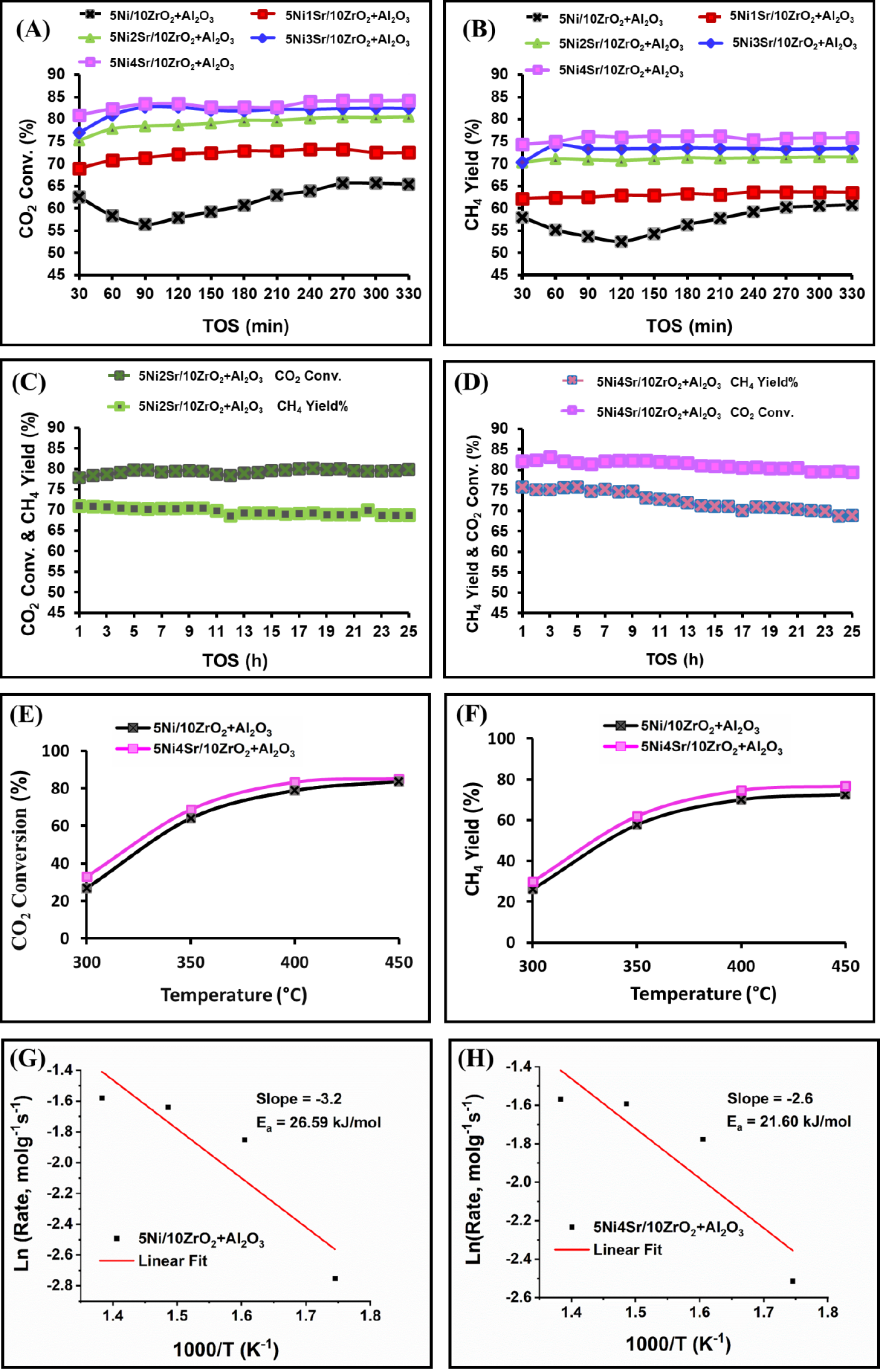
Catalytic activity results of CO_2_: H_2_: Ar (1:4:5 volume ratio) at 6000 ccg^–1^h^–1^ GHSV: (A) CO_2_ conversion % vs 330 min TOS over different catalysts at 400 °C; (B) CH_4_ yield (%) vs 330 min TOS over different catalysts at 400 °C conversion; (C) ‘CO_2_ conversion (%) and CH_4_ yield (%)’ vs long TOS over the Ni2Sr/10ZrO_2_+Al_2_O_3_ catalyst at 400 °C; (D) ‘CO_2_ conversion (%) and CH_4_ yield (%)’ vs long TOS over the Ni2Sr/10ZrO_2_+Al_2_O_3_ catalyst at 400 °C; (E) CO_2_ conversion (%) vs reaction temperature over reduced Ni/10ZrO_2_+Al_2_O_3_ and reduced Ni4Sr/10ZrO_2_+Al_2_O_3_ catalysts; (F) CH_4_ yield (%) vs reaction temperature over reduced Ni/10ZrO_2_+Al_2_O_3_ and reduced Ni4Sr/10ZrO_2_+Al_2_O_3_ catalysts; (G) plot of ln(rate, molg^–1^h^–1^) vs 1000/T (K^–1^) for getting slope and apparent activation energy over 5Ni/10ZrO_2_+Al_2_O_3_; (H) plot of ln(rate, molg^–1^h^–1^) vs 1000/T (K^–1^) for getting slope and apparent activation energy over 5Ni4Sr/10ZrO_2_+Al_2_O_3_.

The catalytic activities of reduced Ni/10ZrO_2_+Al_2_O_3_ and reduced NixSr/10ZrO_2_+Al_2_O_3_ (*x* = 1–4) catalysts are presented in Figure 7. During the reduction process, the active sites ‘Ni’ are derived mostly from ‘moderately interacted NiO-species’ over the catalyst. Along with the formation of active sites, various physio-chemical modifications are also observed during catalyst reduction. In the fresh catalyst system, cubic phases of ZrO_2_ and Al_2_O_3_ are present, while in the reduced catalyst system, intense tetragonal ZrO_2_ and cubic Al_2_O_3_ phases are observed. The tetragonal ZrO_2_ phase is more stable at high temperatures compared to cubic ZrO_2_. Furthermore, the reduced catalyst exhibits a higher intensity of CO_2_-adsorbed species, such as format species, even at atmospheric pressure and normal temperature (confirmed by IR), compared to that of the fresh catalyst sample. The reduced catalyst also possesses weak and moderate strength basic sites, which are constituted by nonreducible surface hydroxyl and nonreducible surface oxide ions. The total amount of acid sites is decreased upon reduction of the 5Ni/10ZrO_2_+Al_2_O_3_ catalyst (confirmed by NH_3_-TPD).

The catalytic activity of reduced Ni/10ZrO_2_+Al_2_O_3_ catalysts showed fluctuations initially but stabilized after 270 min. This catalyst exhibited 65% CO_2_ conversion with 61% CH_4_ yield during the 330 min time on stream at 400 °C reaction temperature. Previous literature has reported a slow decline in CO_2_ conversion for the 20Ni/Al_2_O_3_ catalyst.^18^ Salamony et al. found a rapid drop in CO_2_ conversion from 16% to 0% within 30 min over a zirconia-supported Ni catalyst.^13^ Cai et al. observed the stabilization of catalytic active sites Ni upon incorporation of ZrO_2_ along with the Al_2_O_3_.^19^ They found constant catalytic activity (∼40% CO_2_ conversion) over a Ni-impregnated alumina–zirconia catalyst. In our case, constant catalytic activity was achieved after a 270 min over reduced Ni/10ZrO_2_+Al_2_O_3_ catalysts. Notably, over a reduced Ni/10ZrO_2_+Al_2_O_3_ catalyst, CO was not detected, indicating that the methanation reaction of CO_2_ occurred through a direct pathway (without involving CO in the reaction steps as an intermediate).

The reduced 5NixSr/10ZrO_2_+Al_2_O_3_ (*x* = 1–4) catalysts demonstrated good dispersion of Ni after reduction. After reduction, a noticeable drop in the surface area and pore volume was observed, which is expected for surface modification after the reduction process. A disappearance of SrCO_3_ phases (confirmed by XRD and CO_2_-TPD) and a decrease in concentration of total acidity (confirmed by NH_3_-TPD) were noticed upon reduction of Sr-promoted 5Ni/10ZrO_2_+Al_2_O_3_ catalysts. Upon increasing the Sr loading from 1 to 4 wt % over 5Ni/10ZrO_2_+Al_2_O_3_, a higher density of ‘moderately interacted NiO species’ are cultivated, which facilitates a higher density of active sites ‘Ni’ after reduction. Again, by increasing the addition of Sr up to 4 wt %, the reduced catalyst system exhibited a higher concentration of strong basic sites and a noticeable concentration of moderate-strength acid sites. Upon Sr promotional addition over reduced-Ni/10ZrO_2_+Al_2_O_3_, the constant activity was obtained within 60 min. The CO_2_ conversion () and CH_4_ yield () increased with increasing Sr loading over the 5Ni/10ZrO_2_+Al_2_O_3_ catalyst, with the following trend: 5Ni4Sr/10ZrO_2_+Al_2_O_3_ > 5Ni3Sr/10ZrO_2_+Al_2_O_3_> 5Ni2Sr/10ZrO_2_+Al_2_O_3_ > 5Ni1Sr/10ZrO_2_+Al_2_O_3_> 5Ni/10ZrO_2_+Al_2_O_3_ (. Before dwelling in deep discussion, the major characterization and activity results are summarized in Table 1.

Interestingly, the dispersion of Ni sites over 5Ni1Sr/10ZrO_2_+Al_2_O_3_ and 5Ni3Sr/10ZrO_2_+Al_2_O_3_ catalysts are 1.38 and 1.62 times than the unpromoted catalyst (5Ni/10ZrO_2_+Al_2_O_3_) (Table 1). As per the rise in Ni dispersion, the CO_2_ conversion has also increased up to 72.5% and 82.5% over 5Ni1Sr/10ZrO_2_+Al_2_O_3_ and 5Ni3Sr/10ZrO_2_+Al_2_O_3_, respectively. Upon increasing Sr loading from 1 to 2 wt % and 3 to 4 wt % over 5Ni/10ZrO_2_+Al_2_O_3_, the dispersion of active sites ‘Ni’ does not vary much, but CO_2_ conversion always grows markedly. CO_2_-TPD profile shows that the concentration of strong basic sites is increasing when strontium loading is increased from 1 to 2 wt % and from 3 to 4 wt %. Ni sites initiate hydrogen dissociation, whereas basic sites stabilize the CO_2_-interacted species over the surface. CO_2_-interacted species interact with dissociated hydrogen and undergo a methanation reaction. Now, it is clear that the catalytic activity depends on the dispersion of Ni sites as well as the extent of stabilization of the CO_2_-interacted species.

The long-term stability of the 5Ni2Sr/10ZrO_2_+Al_2_O_3_ and 5Ni4Sr/10ZrO_2_+Al_2_O_3_ catalysts at 400 °C was also studied (Figure 7C,D). The catalytic performance of the 5Ni2Sr/10ZrO_2_+Al_2_O_3_ catalyst is found to be slightly more consistent than that of the 5Ni4Sr/10ZrO_2_+Al_2_O_3_ catalyst. To explain the high CO_2_ conversion (∼80%) and CH_4_ yield (∼70%) observed for up to 28 h in time on stream test through the direct pathway over the 5Ni4Sr/10ZrO_2_+Al_2_O_3_ catalyst, several factors need to be considered. First, the strontium-promoted zirconia–alumina-supported Ni catalyst appears to have the highest density of active sites ‘Ni’ with optimum dispersion. Second, the unique acido-basic profile of the 5Ni4Sr/10ZrO_2_+Al_2_O_3_ catalyst, characterized by the highest concentration of strong basic sites and noticeable concentration of moderate strength acid sites, likely contributes to the enhanced catalytic performance. These specific acid–base sites facilitate the adsorption and activation of CO_2_ and H_2_, whereas the highest density of active sites endorses H_2_ dissociation in time for sequential hydrogenation of formate species into hydroxy methyl → methyl → methane.

The mass transfer limitation over catalyst samples is calculated according to the Mears criterion and Weisz–Prater criterion.^50^ The details of the calculation for external mass transfer limitation and internal mass transfer limitation are shown in Supporting Information S6 and Table S1. Mears criterion for external diffusion is found to be less than 0.15, whereas the Weisz–Prater criterion for internal diffusion is below 1 over each catalyst system. These values confirm the absence of external as well as internal mass transfer limitations over each catalyst system used in this study. The effect of temperature over activity and apparent activation energy for CO_2_ conversion over 5Ni/10ZrO_2_+Al_2_O_3_ and 4 wt % Sr-promoted 5Ni/10ZrO_2_+Al_2_O_3_ catalysts are also studied (Figure 7E,F). CO_2_ conversion and CH_4_ yield increased sharply between 300 and 400 °C and slightly between 400 and 450 °C. The apparent activation energy for CO_2_ conversion over 4 wt % Sr-promoted 5Ni/10ZrO_2_+Al_2_O_3_ catalysts is found to be lower (*E*_a_ = 21.60 kJ/mol) than the nonpromoted 5Ni/10ZrO_2_+Al_2_O_3_ catalyst (*E*_a_ = 26.59 kJ/mol) (Figure 7G,H). Additionally, the stabilization of Ni by incorporating zirconia into the catalyst structure plays a vital role in maintaining the active sites’ integrity and stability during the reaction. Lastly, the large size of Sr^2+^ in the catalyst provides a stabilization capacity for CO_2_-intermediate-like carbonate species. This stabilization effect is crucial for sustaining the reaction and promoting the sequential hydrogenation process that leads to the production of methane. Overall, the exceptional catalytic performance of the 5Ni4Sr/10ZrO_2_+Al_2_O_3_ catalyst in terms of CO_2_ conversion and CH_4_ yield can be attributed to its unique acido-basic profile, presence of highest dense active sites ‘Ni,’ stabilization of Ni through the incorporation of zirconia, and the stabilization capacity of CO_2_-intermediate-like formate species due to the presence of large-sized Sr^2+^ ions. These factors collectively enable the catalyst to efficiently guide the CO_2_ methanation reaction through the direct pathway, leading to high methane production yields.

## 4 Conclusion

The catalytic activity toward CO_2_ methanation was found to depend on the dispersion of active Ni sites (derived from moderately interacted NO species) as well as the extent of stabilization of CO_2_-surface intermediate species. Upon 1 wt % loading of Sr over 5Ni/10ZrO_2_+Al_2_O_3_, the Ni dispersion over the catalyst surface grows in comparison to the unpromoted catalyst, whereas upon 2 wt % Sr loading, dispersion of Ni is not affected much, but the concentration of strong basic sites is increased markedly. Again, at 3 wt % Sr loading over 5Ni/10ZrO_2_+Al_2_O_3_, dispersion of Ni sites increases (in comparison to 5Ni2Sr/10ZrO_2_+Al_2_O_3_), while at 4 wt % Sr loading, concentration of strong basic sites increases significantly. The Sr-promoted 5Ni/10ZrO_2_+Al_2_O_3_ catalyst has a lower apparent activation energy for CO_2_ conversion than the unpromoted catalyst. The unique acido-basic profiles, characterized by strong basic and moderate acid sites, facilitate the sequential hydrogenation of format species into hydroxy methyl → methyl → methane. The 5Ni4Sr/10ZrO_2_+Al_2_O_3_ catalyst demonstrates exceptional performance, achieving approximately 80% CO_2_ conversion and 70% CH_4_ yield for up to 25 h time on stream via the direct methanation pathway. In summary, the combination of zirconia–alumina support, Ni catalyst, and Sr promotion proves to be a highly efficient and stable system for CO_2_ methanation, opening up new possibilities for CO_*x*_-free CH_4_ production with potential applications in addressing environmental concerns and energy sustainability.

## Supporting Information Available

Figure S1: XRD pattern of 5Ni2Sr/10ZrO_2_+Al_2_O_3._ Figure S2: Nitrogen sorption isotherm of fresh and reduced 5NixSr/10ZrO_2_+Al_2_O_3_ (*x* = 0–4 wt %) catalyst system. Figure S3: Infrared spectra of fresh and reduced 5NixSr/10ZrO_2_+Al_2_O_3_ (*x* = 0–4 wt %) catalyst system. Figure S4: CO_2_-TPD profile over (A) fresh 5Ni/10ZrO_2_+Al_2_O_3_, (B) reduced 5Ni/10ZrO_2_+Al_2_O_3_O_2_, (C) fresh 5Ni1Sr/10ZrO_2_+Al_2_O_3_ catalyst and reduced 5Ni1Sr/10ZrO_2_+Al_2_O_3_ catalyst, (D) fresh 5Ni2Sr/10ZrO_2_+Al_2_O_3_ catalyst and reduced 5Ni2Sr/10ZrO_2_+Al_2_O_3_ catalyst, (E) fresh 5Ni3Sr/10ZrO_2_+Al_2_O_3_ catalyst and reduced 5Ni3Sr/10ZrO_2_+Al_2_O_3_ catalyst, (F) fresh 5Ni4Sr/10ZrO_2_+Al_2_O_3_ catalyst and reduced 5Ni4Sr/10ZrO_2_+Al_2_O_3_catalyst. Figure S5; NH_3_-TPD profile over (A) fresh 5NixSr/10ZrO_2_+Al_2_O_3_ (*x* = 0–4 wt %) catalyst, (B) fresh 5Ni/10ZrO_2_+Al_2_O_3_ and reduced 5Ni/10ZrO_2_+Al_2_O_3_O_2_, (C) fresh 5Ni1Sr/10ZrO_2_+Al_2_O_3_ catalyst and reduced 5Ni1Sr/10ZrO_2_+Al_2_O_3_ catalyst, (D) fresh 5Ni2Sr/10ZrO_2_+Al_2_O_3_ catalyst and reduced 5Ni2Sr/10ZrO_2_+Al_2_O_3_ catalyst, (E) fresh 5Ni3Sr/10ZrO_2_+Al_2_O_3_ catalyst and reduced 5Ni3Sr/10ZrO_2_+Al_2_O_3_ catalyst, (F) fresh 5Ni4Sr/10ZrO_2_+Al_2_O_3_ catalyst and reduced 5Ni4Sr/10ZrO_2_+Al_2_O_3_ catalyst. Figure S6: Calculation of Mass transfer limitation. Table S7: Rate of CO_2_ conversion and Mears criterion for external diffusion limitation and Weisz criterion for internal diffusion limitation (PDF)
