# Supplementary material for: Impact of Sr Addition on Zirconia–Alumina-Supported Ni Catalyst for COx-Free CH4 Production via CO2 Methanation
Source: ACS Omega. 2024 Feb 14;9(8):9309–20. doi: 10.1021/acsomega.3c08536 (PMC10905718; doi:10.1021/acsomega.3c08536)
Supplement: Supplementary file 1 — ao3c08536_si_001.pdf [file ao3c08536_si_001.pdf]

Supporting Information

Impact of Sr addition on Zirconia-alumina Supported Ni Catalyst for CO_x Free CH_4 Production via CO_2 Methanation

Abdulaziz A.M. Abahussain[1] , Ahmed S. Al-Fatesh[1*], Yuvrajsinh B. Rajput[2], Ahmed I. Osman[3]*, Salwa B. Alreshaidan[4] , Hamid Ahmed[1], Anis H. Fakeeha[1], Abdulrhman S. Al-Awadi[1], Radwa A. El-Salamony[5], Rawesh Kumar[2*]

[1]Chemical Engineering Department, College of Engineering, King Saud University, P.O. Box 800, Riyadh 11421, Saudi Arabia.

[2]Department of Chemistry, Indus University, Ahmedabad, Gujarat,382115, India.

[3]School of Chemistry and Chemical Engineering, Queen's University Belfast, Belfast, BT9 5AG, Northern Ireland, UK

[4]Chemical Engineering Department, College of Engineering, King Saud University, P.O. Box 800, Riyadh 11421, Saudi Arabia

[5]Process Development Department, Egyptian Petroleum Research Institute (EPRI), Cairo 11727, Egypt

*Correspondence: aalfatesh@ksu.edu.sa; aosmanahmed01@qub.ac.uk;

kr.rawesh@gmail.com

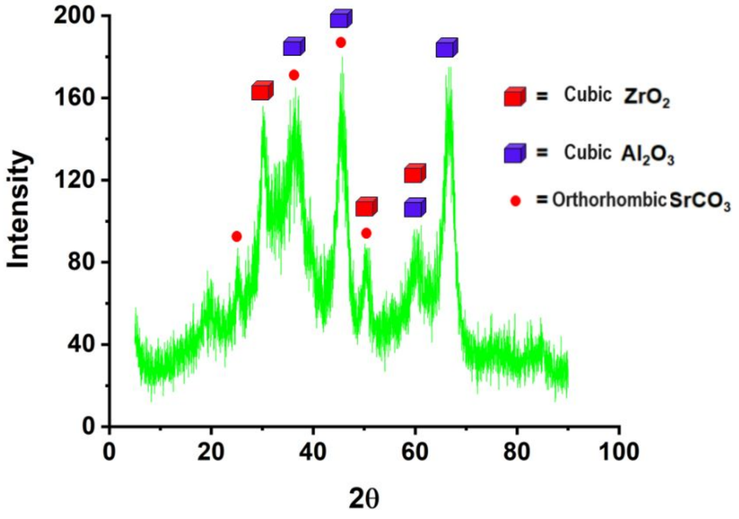

Figure S1 XRD pattern of $5Ni2Sr/10ZrO_2+Al_2O_3$

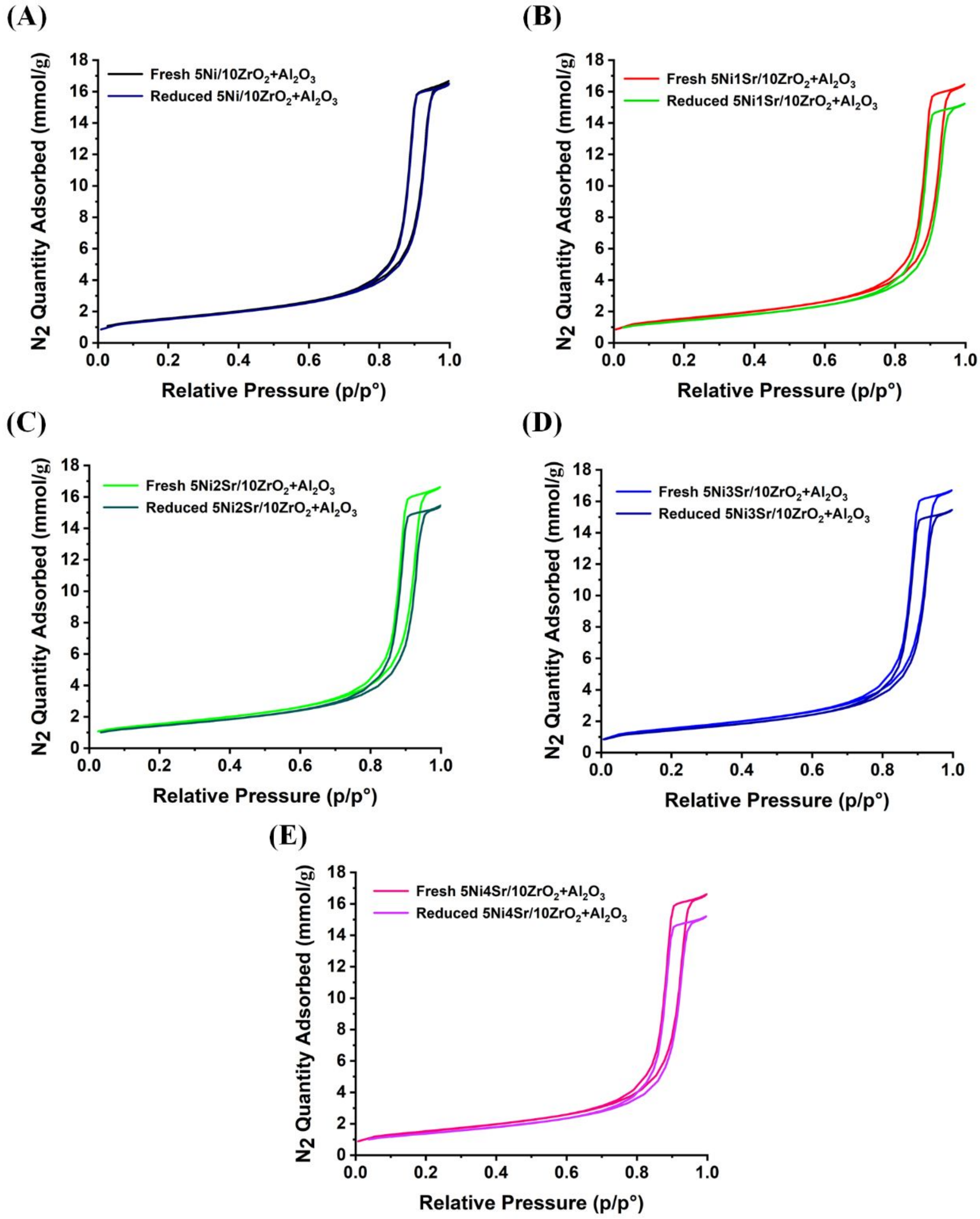

Figure S2. Nitrogen sorption isotherm of fresh and reduced catalyst (A) 5Ni/10ZrO_2+Al_2O_3 (B) 5Ni1Sr/10ZrO_2+Al_2O_3 (C) 5Ni2Sr/10ZrO_2+Al_2O_3(D) 5Ni3Sr/10ZrO_2+Al_2O_3 (E) 5Ni4Sr/10ZrO_2+Al_2O_3

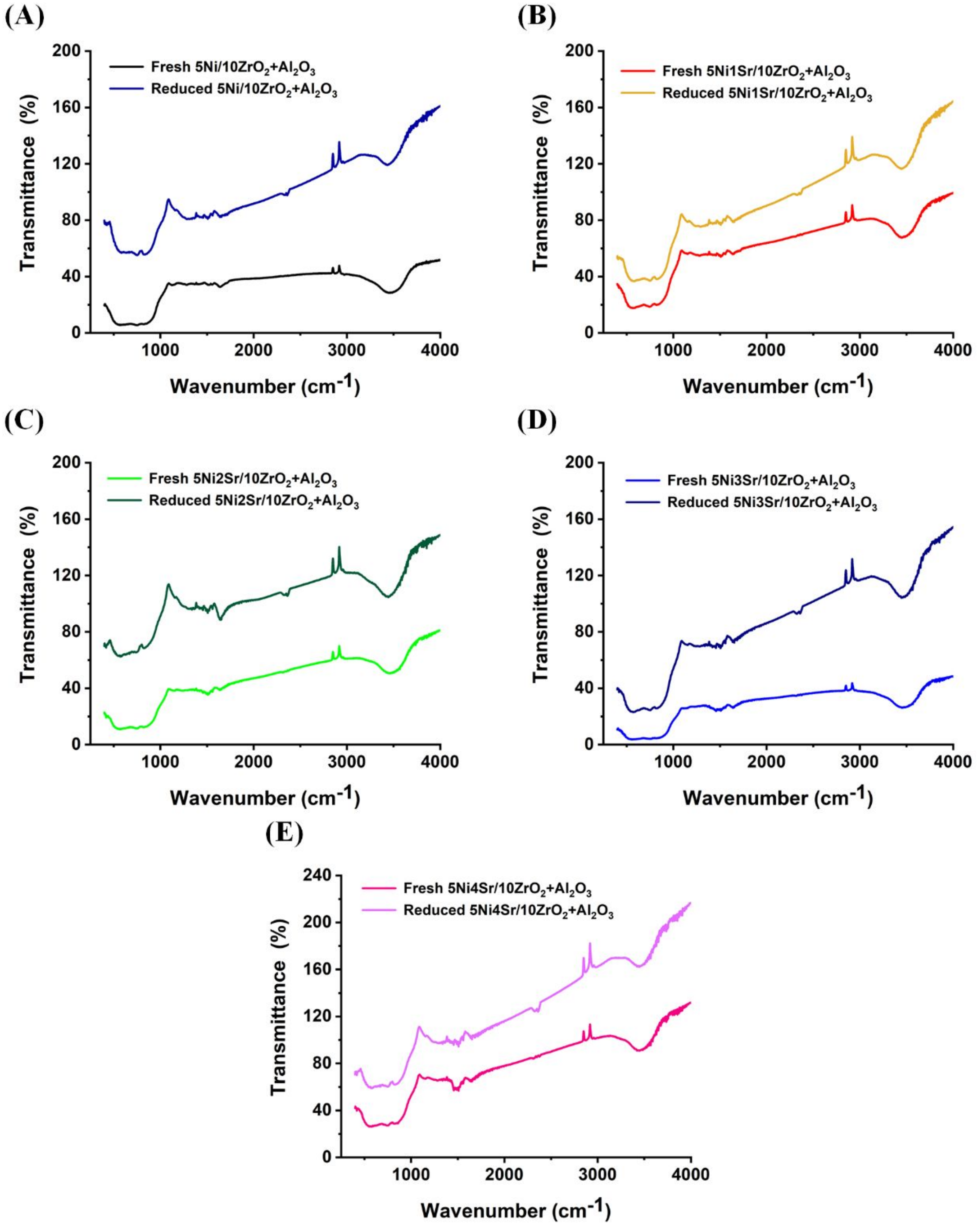

Figure S3 Infrared spectra of fresh and reduced catalysts (A) $5Ni/10ZrO_2+Al_2O_3$ (B) $5Ni1Sr/10ZrO_2+Al_2O_3$ (C) $5Ni2Sr/10ZrO_2+Al_2O_3$(D) $5Ni3Sr/10ZrO_2+Al_2O_3$ (E) $5Ni4Sr/10ZrO_2+Al_2O_3$

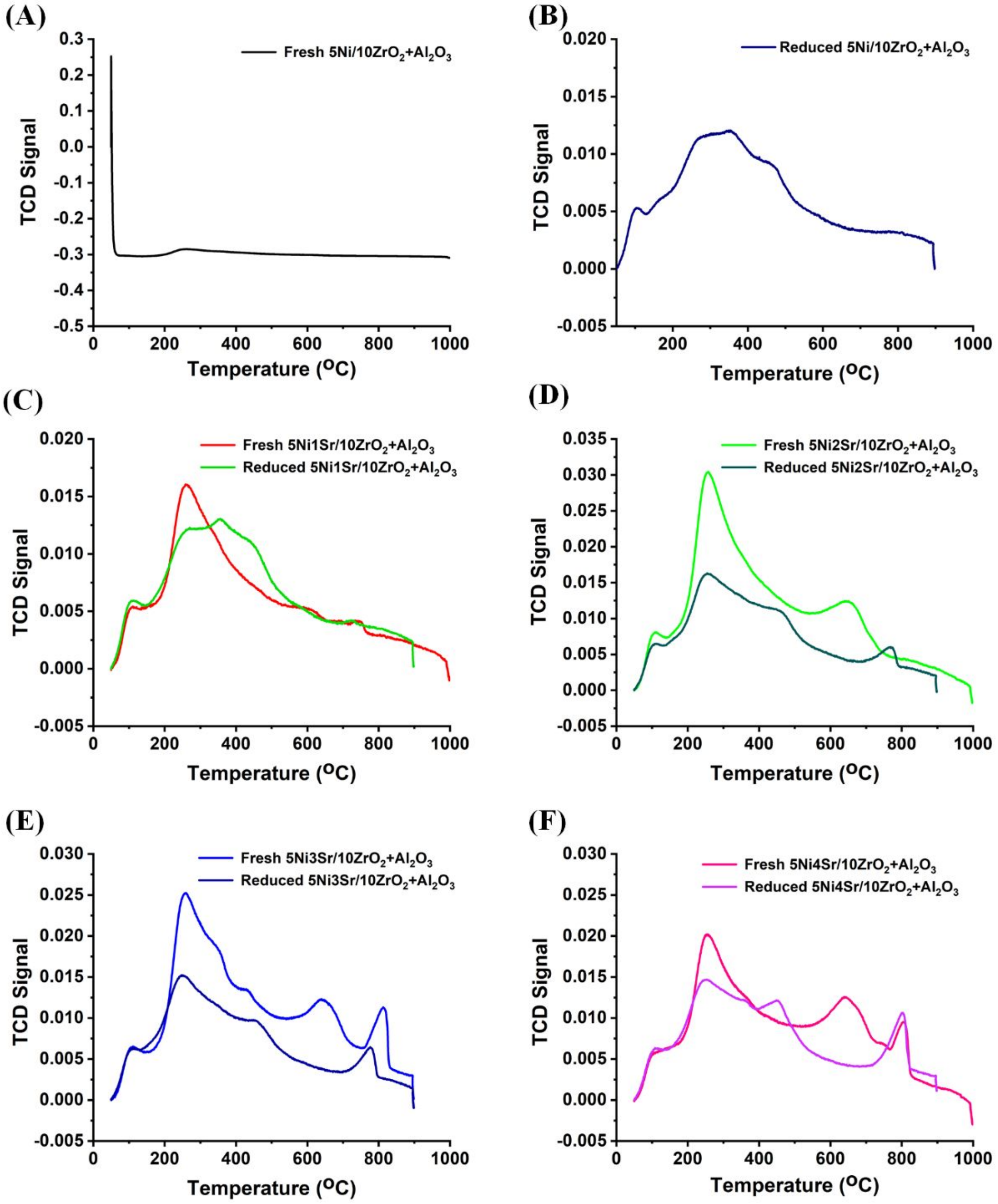

Figure S4. CO_2-TPD profile over (A) Fresh-5Ni/10ZrO_2+Al_2O_3 (B) Reduced-5Ni/10ZrO_2+$Al_2O_3O_2$ (C) Fresh-5Ni1Sr/10ZrO_2+Al_2O_3 catalyst and Reduced- 5Ni1Sr/10ZrO_2+Al_2O_3 catalyst (D) Fresh-5Ni2Sr/10ZrO_2+Al_2O_3 catalyst and Reduced-5Ni2Sr/10ZrO_2+Al_2O_3 catalyst (E) Fresh-5Ni3Sr/10ZrO_2+Al_2O_3 catalyst and Reduced-5Ni3Sr/10ZrO_2+Al_2O_3 catalyst (F)) Fresh-5Ni4Sr/10ZrO_2+Al_2O_3 catalyst and Reduced-5Ni4Sr/10ZrO_2+Al_2O_3 catalyst

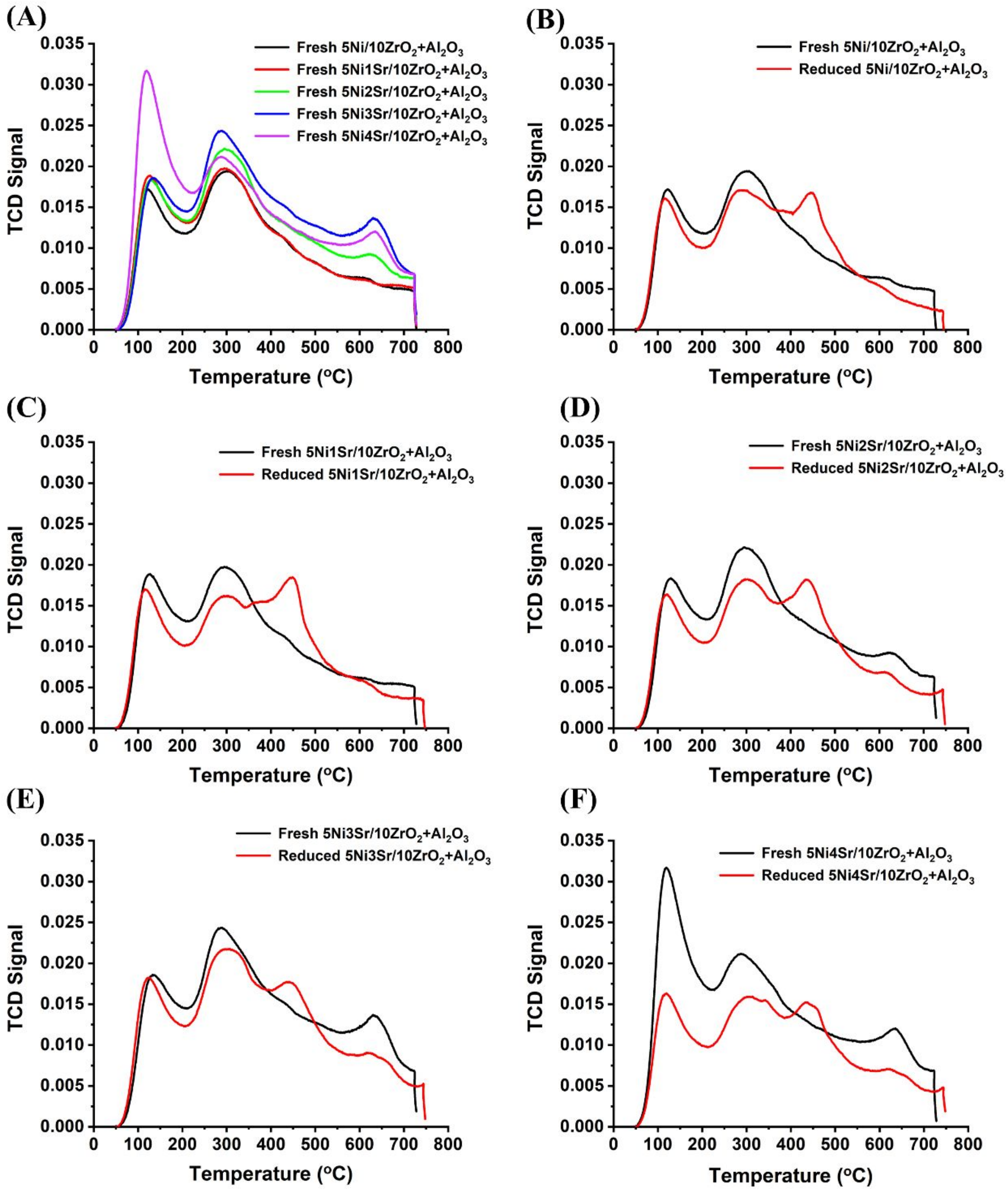

Figure S5. NH_3-TPD profile over (A) Fresh-5NixSr/10ZrO_2+Al_2O_3 (x = 0-4 wt%) catalyst (B) Fresh-5Ni/10ZrO_2+Al_2O_3 and Reduced-5Ni/10ZrO_2+$Al_2O_3O_2$ (C) Fresh-5Ni1Sr/10ZrO_2+Al_2O_3 catalyst and Reduced-5Ni1Sr/10ZrO_2+Al_2O_3 catalyst (D) Fresh-5Ni2Sr/10ZrO_2+Al_2O_3 catalyst and Reduced-5Ni2Sr/10ZrO_2+Al_2O_3 catalyst (E) Fresh-5Ni3Sr/10ZrO_2+Al_2O_3 catalyst and Reduced-5Ni3Sr/10ZrO_2+Al_2O_3 catalyst (F) Fresh-5Ni4Sr/10ZrO_2+Al_2O_3 catalyst and Reduced-5Ni4Sr/10ZrO_2+Al_2O_3 catalyst

S6. Calculation of Mass transfer limitation:

The absence of external mass transfer limitations was checked [4] by the Mears criterion, $-r_A'\rho_b R\, n / (k_c C_{Ab}) < 0.15$, where $-\mathbf{r_A'}$ is the measured reaction rate in $\mathbf{k_{mol} kg_{cat}^{-1}\, s^{-1}}$, $\boldsymbol{\rho_b}$ is the bulk density of catalyst bed in kg m^{-3}, **R** is the catalyst particle radius in meter, **n** is the reaction order, k_c is the mass transfer coefficient in $\mathbf{m\ s^{-1}}$ and $\mathbf{C_{Ab}}$ is the bulk gas concentration of A in kmol m^{-3}. In the present work, CH_4 conversion rate ($-r_{CH_4}$**) and CO_2 conversion rate** (–

r_{CO_2}), is in $\mathbf{k_{mol}kg_{cat}^{-1}\ s^{-1}}$, average solid catalyst density (**ρc**) is 4000 $\mathbf{kgm^{-3}}$, average density of catalyst (ρ_b) is 2000 $\mathbf{kg\ m^{-3}}$ ($\boldsymbol{\rho_b=(1-\phi_b)\rho_c}$), bed porosity (**$\phi_b$**) is taken as 0.38, average radius (R) is 7.50 x 10^{-5} m, order of reaction (n) is 2 and **C_{Ab}, is** bulk gas average concentration (in **kmol m^{-3}**) over 5Ni/ZrO_2+Al_2O_3 and 5NixSr/ZrO_2+Al_2O_3 (x = 1-4 wt%) catalyst. A conservative estimate of mass transfer coefficient (k_c) is estimated by using Dwivedi and Upadhyay correlation, which are typically encountered in laboratory reactors such as the one used in the present study. The absence of internal mass transfer limitations was ascertained by means of the Weisz–Prater criterion, $C_{WP} = -r_A\rho_c R^2 /(D_eC_{As}) < 1$). In this criterion, the Gas concentration at catalyst surface C_{As} is assumed to be equal to C_{Ab} ($C_{As}= C_{Ab}$) and effective diffusibility De is taken as = $D_{AB}.\ \phi_p.\sigma_c/\tau$; where D_{AB} is the Molecular diffusion of CO_2 and H_2, ϕ_p is pellet porosity, σ_c is the constriction factor, and τ is tortuosity. ϕ_p, σ_c and τ have values of 0.38, 0.8 and 3, respectively. These numbers resulted in a value for the Weisz–Prater criterion of < 1. The Mears criterion and Weisz-Prater criterion values for 5Ni/ZrO_2+Al_2O_3 and 5NixSr/ZrO_2+Al_2O_3 (x = 1-4 wt%) catalyst mentioned in the Table below for external and internal mass transfer, respectively. The absence of external and internal mass transfer limitations is found for all catalytic systems used in the present case.

Table S1. Rate of CO_2 conversion and Mears criterion for external diffusion limitation and Weisz criterion for internal diffusion limitation

Catalyst	Surface Area (m^2/g)	CO_2 Conversion (%)	Rate of CO_2 conversion (mol_{CO_2}/g_{cat}/h)	Mears criterion for external diffusion concerning CO_2	Weisz criterion for internal diffusion concerning CO_2
5Ni/ ZrO_2+Al_2O_3	121.25	65	-0.1594	1.98 x 10^{-03}	3.72 x 10^{-03}
5Ni1Sr/ ZrO_2+Al_2O_3	113.54	72.5	-0.1778	2.21 x 10^{-03}	4.15 x 10^{-03}
5Ni2Sr/ ZrO_2+Al_2O_3	114.23	80.6	-0.1977	2.46 x 10^{-03}	4.61 x 10^{-03}
5Ni3Sr/ ZrO_2+Al_2O_3	114.26	82.5	-0.2023	2.52 x 10^{-03}	4.72 x 10^{-03}
5Ni4Sr/ ZrO_2+Al_2O_3	110.97	84.3	-0.2067	2.57 x 10^{-03}	4.82 x 10^{-03}
